# Resistance Exercise Associated with Camu-Camu (*Myrciaria dubia*) and Creatine Supplementation Modulates Antioxidant Response and Cardiac Parameters in Wistar Rats

**DOI:** 10.3390/nu17223587

**Published:** 2025-11-17

**Authors:** Thaís Cupertino Fialho, Lívia Carvalho Sette Abrantes, Karina Vitória Cipriana Martins, Renner Philipe Rodrigues Carvalho, Camilo José Ramírez-López, Alex Filipe Ramos de Sousa, Luiz Otávio Guimarães-Ervilha, Lívya Alves Oliveira, Gabrieli Fernandes Lacerda, Ana Júlia Brandão Moreira, Sebastião Felipe Ferreira Costa, Valéria Silva de Lana, Mariana Machado-Neves, Antônio José Natali, Pedro Forte, Luciano Bernardes Leite, Izabela Maria Montezano Carvalho, Hércia Stampini Duarte Martino, Renê Chagas da Silva, Ceres Mattos Della Lucia

**Affiliations:** 1 Department of Nutrition and Health, Universidade Federal de Viçosa, Av. Peter Henry Rolfs, s/n-Campus Universitário, Viçosa 36570-900, MG, Brazil; thais.cupertino@ufv.br (T.C.F.); livia.abrantes@ufv.br (L.C.S.A.); karina.cipriana@ufv.br (K.V.C.M.); livya.oliveira@ufv.br (L.A.O.); gabrieli.lacerda@ufv.br (G.F.L.); valeria.lana@ufv.br (V.S.d.L.); izabela.carvalho@ufv.br (I.M.M.C.); hercia@ufv.br (H.S.D.M.); 2 Department of General Biology, Universidade Federal de Viçosa, Av. Peter Henry Rolfs, s/n-Campus Universitário, Viçosa 36570-900, MG, Brazil; renner.carvalho@ufv.br (R.P.R.C.); camilo.lopez@ufv.br (C.J.R.-L.); alex.sousa@ufv.br (A.F.R.d.S.); luiz.ervilha@ufv.br (L.O.G.-E.); mariana.mneves@ufv.br (M.M.-N.); 3 Department of Physical Education, Universidade Federal de Viçosa, Av. Peter Henry Rolfs, s/n-Campus Universitário, Viçosa 36570-900, MG, Brazil; ana.j.moreira@ufv.br (A.J.B.M.); sebastiao.costa@ufv.br (S.F.F.C.); anatali@ufv.br (A.J.N.); luciano.leite@ufv.br (L.B.L.); 4 Department of Sports, Instituto Politécnico de Bragança, 5300-253 Bragança, Portugal; pedromiguelforte@gmail.com; 5 Research Center for Active Living and Wellbeing (Livewell), Instituto Politécnico de Bragança, 5300-253 Bragança, Portugal; 6 Department of Sports, Higher Institute of Educational Sciences of the Douro, 4560-708 Penafiel, Portugal; 7 Department of Physics, Universidade Federal de Viçosa, Av. Peter Henry Rolfs, s/n-Campus Universitário, Viçosa 36570-900, MG, Brazil; rene.silva@ufv.br

**Keywords:** resistance training, bioactive compounds, antioxidants, supplements, oxidative stress, experimental study

## Abstract

Background: Resistance exercise (RE) is recognized for promoting the development of muscle strength and mass, as well as contributing positively to cardiovascular health. The combination of this type of exercise with the intake of foods rich in bioactive compounds, such as camu-camu (*Myrciaria dubia*), and creatine supplementation may be an interesting strategy to enhance the cardiovascular system. Objective: This study aimed to evaluate the effects of RE and supplementation with camu-camu and creatine on oxidative balance, mineral content, ATPase enzyme activity, and histological changes in the heart of Wistar rats. Methods: Forty-eight adult rats were divided into eight groups, with or without RE. The groups received a control diet (AIN-93M), camu-camu (200 mg/kg/day), creatine (300 mg/kg for 7 days and 50 mg/kg/day thereafter), or a combination of both. The RE protocol was performed on a vertical ladder three times a week for eight weeks. At the end, the animals were anesthetized and euthanized for tissue collection. Results: The trained control group that received a standard diet (AIN-T) showed greater activity of superoxide dismutase and catalase. The trained group receiving creatine and camu-camu supplementation (CC + Cr-T) showed higher total antioxidant capacity (FRAP), increased Mg^2+^-ATPase activity, higher nitric oxide levels, and a greater diameter of cardiac muscle fibers. No pathological changes were observed in heart histology in any group, indicating preservation of tissue integrity. Conclusions: RE associated with camu-camu and creatine supplementation may be an effective strategy for modulating antioxidant and functional aspects of the heart.

## 1. Introduction

Resistance exercise (RE), characterized by muscle contractions against an external load, promotes physiological adaptations in both skeletal and cardiac muscle, demonstrating a functional interaction between these systems in response to physical training [1,2]. Skeletal muscle, which accounts for approximately 40–50% of total body mass, responds to training through increased synthesis of contractile proteins and muscle fiber hypertrophy [3,4]. These processes are regulated by factors such as training intensity, nutrient availability, hormonal profile, and activation of specific signaling pathways [3,5,6]. In addition to increasing or maintaining muscle mass and strength, RE also contributes to the prevention of cardiovascular disease and the reduction in related risk factors, reinforcing its role as a therapeutic and preventive strategy [2].

Exercise induces cardiovascular adaptations characterized by physiological changes in response to increased hemodynamic demand. Cardiac hypertrophy resulting from this stimulus is considered a beneficial adaptive response, provided it remains within physiological limits [7]. In the context of RE, there is myocardial strengthening, enhanced contractility, and greater efficiency of cardiac muscle fibers, which improve the heart’s pumping capacity [7,8]. These adaptations also contribute to lower blood pressure and overall improvement in cardiac function, acting preventively against cardiovascular disease [2,9].

The combination of physical exercise and nutritional strategies has been explored to maximize training benefits. Among these strategies, camu-camu (*Myrciaria dubia*), an Amazonian fruit with high nutritional value, stands out for its high vitamin C content and phenolic compounds, particularly anthocyanins [10,11,12]. This composition provides anti-allergic, antioxidant, and anti-inflammatory properties, as well as benefits for intestinal health [13,14,15], which may enhance the effects of RE and influence cardiac parameters [16]. Moreover, camu-camu contains minerals such as iron, phosphorus, potassium, and calcium, the latter two being essential for cardiac contractility and excitability, processes regulated by ATPase pump activity [17,18,19,20,21,22].

When used under professional guidance, dietary supplements can further enhance health benefits. In this context, creatine is notable. It is one of the most extensively studied supplements and is recognized for improving strength and endurance performance [23,24]. Studies also suggest that creatine supplementation may reduce oxidative stress and have beneficial effects on cardiovascular health by supporting myocardial bioenergetics and through its antioxidant action [23,25,26].

Despite the growing evidence supporting the isolated benefits of both camu-camu and creatine, no previous studies have investigated their combined use in the context of RE. This gap is relevant, as creatine and phenolic compounds may act synergistically to modulate oxidative balance and cardiac adaptations to training. Based on the antioxidant potential of camu-camu and creatine, as well as cardioprotective effects attributed to RE, we hypothesize that the combination of these interventions will reduce oxidative stress and preserve cardiac morphology. Therefore, this study aims to investigate the effects of RE and supplementation with camu-camu and creatine on oxidative balance, mineral content, ATPase enzyme activity, and histological changes in the heart of Wistar rats.

## 2. Materials and Methods

### 2.1. Camu-Camu and Creatine

Powdered camu-camu (obtained from fruit pulp) and creatine were commercially purchased and stored at room temperature in a dry, well-ventilated area, protected from direct sunlight. The selection of creatine was based on the degree of purity to ensure the quality and reliability of the product used.

### 2.2. Proximate Composition of Camu-Camu

The proximate composition was determined using standardized methods, according to AOAC [27]. Moisture content was determined using the gravimetric method, total lipids by Soxhlet extraction, ash content by incineration in a muffle furnace, protein content by the Kjeldahl method, carbohydrates were calculated by difference, and dietary fiber was analyzed using the enzymatic-gravimetric method.

### 2.3. Estimation of Total Phenolic Concentration

Phenolic compounds were extracted from powdered camu-camu according to the method described by Bloor [28], using 60% methanol as solvent. The samples were stirred at 180 rpm for 15 min at room temperature and then centrifuged at 3500 rpm for 5 min. The supernatant was collected for further analysis. The concentration of total phenolics was determined by the Folin–Ciocalteu method, according to Singleton et al. [29]. The reactions were performed in test tubes containing extract, 20% Folin–Ciocalteu reagent, and 7.5% sodium carbonate, with absorbance readings at 760 nm after 30 min of reaction. The results were expressed in mg of gallic acid equivalents per gram of sample (mg GAE/g).

### 2.4. Antioxidant Actizvity

#### 2.4.1. Determination of Antioxidant Capacity by the 2,2-diphenyl-1-picrylhydrazyl (DPPH) Radical Method

Antioxidant activity was determined using the DPPH radical method, according to Bloor [28], using extracts obtained with methanol: water (60:40, *v*/*v*). The samples were shaken for 25 min, centrifuged at 3500 rpm for 5 min, filtered, and stored in amber vials. For the analysis, 100 μL of the extract were mixed with 1500 μL of a methanolic DPPH solution and incubated for 30 min in a light-protected environment. Absorbance was measured at 517 nm using a spectrophotometer with 96-well plates. Results were expressed as millimoles of Trolox equivalents per gram of sample (mM Trolox/g).

#### 2.4.2. Trolox Equivalent Antioxidant Capacity (TEAC) Assay

Antioxidant capacity was determined using the TEAC method, as described by Leite-Legatti et al. [30]. Extracts were obtained from 1 g of powdered camu-camu, diluted in 50% ethanol, and submitted to ultrasonic bath for 10 min, followed by centrifugation at 5000 rpm for 10 min at 5 °C. The ABTS^+^• radical was generated by reacting ABTS with potassium persulfate and diluted to an absorbance of 0.70 ± 0.02 at 734 nm. The reaction between 250 μL of the ABTS^+^• solution and 50 μL of the extract was conducted for 6 min in the dark at room temperature. Absorbance was measured at 734 nm using a microplate reader. Results were expressed as micromoles of Trolox equivalent per gram of sample (μmol TE/g), and the percentage of ABTS^+^• radical inhibition was calculated based on the reduction in absorbance.

#### 2.4.3. Ferric Reducing Antioxidant Power (FRAP) Assay

Antioxidant capacity by the FRAP method was evaluated according to Oyaizu [31], with adaptations. The FRAP reagent was prepared using 0.3 M acetate buffer, 10 mM TPTZ, and 20 mM ferric chloride. The reaction was carried out with 20 μL of the extract and incubated at 37 °C for 30 min. Absorbance was measured at 595 nm using a microplate reader. Results were expressed as micromoles of Trolox equivalent per gram of sample (μmol TE/g).

### 2.5. Ascorbic Acid Quantification

The extraction and analysis of ascorbic acid were performed according to the optimized protocol by Campos et al. [32]. Three grams of powdered camu-camu were weighed and extracted with 15 mL of a solution containing 3% metaphosphoric acid, 8% acetic acid, 0.6 mol/L sulfuric acid, and 1 mM EDTA. After centrifugation at 3000× *g* for 10 min, the supernatant was vacuum-filtered using a Büchner funnel, and the volume was adjusted to 25 mL with ultrapure water. Prior to analysis, the extract was filtered through 0.22 µm membranes (HV Millex, Burlington, MA, USA).

Ascorbic acid quantification was performed by high-performance liquid chromatography (HPLC) using a Shimadzu system (SCL 10A VP) with an RP-18 column (250 mm × 4 mm, 5 µm). The mobile phase consisted of a 1 mM NaH_2_PO_4_ solution with 1 mM EDTA, pH 3.0, at a flow rate of 1.0 mL/min. Detection was carried out at 245 nm using a diode array detector (Shimadzu SOD-M10 AVP, Kyoto, Japan). Identification and quantification were based on retention time and UV spectra compared to an L-ascorbic acid standard, with calibration curves constructed from different concentrations of the standard solution.

### 2.6. Mineral Content

The samples were dried and weighed for homogenization, then stored in previously decontaminated glass bottles. For the digestion of chemical elements, a mixture of nitric and perchloric acids (4:1) was applied to 0.5 g of the sample. The digestion was carried out in a fume hood with progressive heating up to 200 °C, until the extract acquired a crystalline appearance. After cooling, the volume was adjusted to 25 mL with deionized water.

Next, the mineral content was analyzed by inductively coupled plasma optical emission spectrometry (ICP-OES), using equipment calibrated with a multi-element standard solution and certified reference material. This technique allows quantification of elements based on the light emission from atoms excited in the plasma.

### 2.7. Animals and Experimental Diets

Forty-eight young adults male Wistar rats (*Rattus norvegicus*, albinus variety, Rodentia), aged 12 weeks, from the Central Animal Facility of the Center of Biological and Health Sciences, Universidade Federal de Viçosa, Brazil, with an average initial weight of 250 g, were used. The sample calculation was performed as proposed by Fontelles et al. [33]. Additionally, a sample power analysis was performed using SOD activity as the primary variable. Considering η^2^ = 0.70 (f = 1.53), α = 0.05, n = 6 per group, and eight experimental groups, the statistical power (1 − β) was 0.99, indicating adequate sensitivity to detect differences among groups. The analysis was performed using the G*Power software, version 3.1.

**Figure 1:**
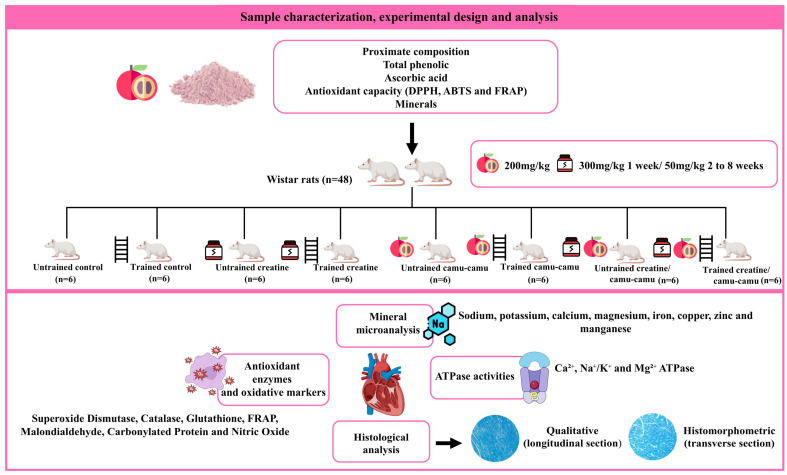
Flowchart of the sample composition, experimental design and main analyses.

The animals were randomly divided by weight into eight experimental groups with 6 animals each (n = 6/group), as follows: (i) the untrained control group receiving a standard diet (AIN-NT/n = 6); (ii) the trained control group receiving a standard diet (AIN-T/n = 6); (iii) the untrained group receiving creatine supplementation (CR-NT/n = 6); (iv) the trained group receiving creatine supplementation (CR-T/n = 6); (v) the untrained group receiving camu-camu supplementation (CC-NT/n = 6); (vi) the trained group receiving camu-camu supplementation (CC-T/n = 6); (vii) the untrained group receiving creatine and camu-camu supplementation (CC + Cr-NT/n = 6); and (viii) the trained group receiving creatine and camu-camu supplementation (CC + Cr-T/n = 6) (Figure 1).

The animals remained in an acclimatization period for 4 weeks before the interventions began. The experimental diet, formulated and produced according to the AIN-93M recommendations proposed by the American Institute of Nutrition [34], was introduced and maintained for 8 weeks (Table 1). The animals received water ad libitum and a portioned diet daily. The diets were normocaloric, normoproteic, and normolipidic. The animals were housed individually in stainless steel cages in an environment with controlled temperature (22 °C ± 2 °C) and an automatically controlled 12 h light/dark cycle.

Regarding supplementation, creatine and camu-camu were added to the diet at doses selected based on previous studies with similar outcomes that demonstrated efficacy and safety in rodents and calculated according to the animals’ body weight (mg/kg) to ensure accurate individual intake. The creatine dosage used was 300 mg/kg of the animal’s body weight per day in the form of creatine monohydrate during the first week, followed by 50 mg/kg of body weight per day until the end of the experiment [35,36,37]. Camu-camu was administered at a dose of 200 mg/kg of the animal’s body weight per day in powder form [38,39,40].

### 2.8. Resistance Exercise Training

The RE protocol was based on the method described by Hornberger and Farrar [41], with adaptations. All animals underwent a two-week familiarization period, with five sessions per week. During this period, they climbed a vertical ladder (height: 1.1 m; width: 0.18 m; rung spacing: 2 cm; inclination: 80°) with an external load attached to the tail. After familiarization, the maximum load test (MLT) was performed. The test started with a load corresponding to 50% of the animal’s body weight, followed by 75%, 90%, and 100%. Additional increments of 30 g were then added until the animal reached exhaustion. Between each attempt, the animals rested for 120 seconds. This procedure was repeated every 15 days over the course of 8 weeks for all experimental groups.

After the MLT, the animals in the trained groups began performing RE three times per week. Each session consisted of 4 to 9 ladder climbs with progressive loads of 50%, 75%, 90%, and 100% of the previously determined maximal load, followed by additional 30 g increments until completing 9 climbs. A 120 s rest interval was maintained between climbs. Thus, training loads were adjusted every 15 days based on the animals’ performance in previous MLT sessions, with increments expressed as a percentage of the new maximum load, ensuring progressive overload. The tails of the animals were protected with soft adhesive tape to prevent injuries during load attachment.

Handling of the animals was carried out by the same trained personnel at approximately the same times each day to minimize stress and reduce variability in spontaneous activity. Furthermore, the untrained groups performed a weekly vertical stair climbing session, without additional load, to standardize exposure to the training environment. These measures were implemented to ensure uniform exposure to experimental conditions and comparability between groups.

### 2.9. Euthanasia

At the end of the 8-week experimental period, 48 h after the last maximal load test and after a 6 h fast, the animals were anesthetized with isoflurane (Isoforine, Cristália^®^, Itapira, Brazil) and euthanized by cardiac puncture. A portion of the left ventricle was frozen in liquid nitrogen and stored at −80 °C until analysis. Another portion was fixed in formalin for later histological evaluation.

### 2.10. Ethical Aspects

All procedures were conducted in accordance with ethical principles of animal experimentation. The study was approved by the Ethics Committee on Animal Use of the Federal University of Viçosa (CEUA/UFV), under protocol number 08/2024, dated 15 May 2024.

### 2.11. Murinometric Measurements and Food Intake Assessment

The body weight of the animals was evaluated weekly (Mettler Toledo^®^, Barueri, SP, Brazil), and weight gain or loss was determined by the difference between the final and initial values. Weekly food intake (in grams) was calculated by subtracting the amount of leftover feed in the feeders, along with any spillage collected from the bottom of the cages, from the total amount of feed offered.

Abdominal circumference was measured at the beginning and end of the experiment at the midpoint between the skull and the pelvic limbs, as described by Pereira et al. [42].

The body mass index (BMI) was calculated as the ratio between body weight (g) and the square of the naso-anal length (cm^2^), according to Novelli et al. [43].

The feed efficiency coefficient (FEC) was calculated using the formula: (final weight gain/total food intake) × 100, as described by Hegsted [44].

### 2.12. Oxidative Stress Analysis

Left ventricle samples (100 mg) were weighed and homogenized in 1 mL of phosphate buffer (0.1 M, pH 7.4). After homogenization, the samples were centrifuged at 10,000× *g* for 10 min at 4 °C. Protein concentration in the supernatant was quantified using the Bradford method, and the values were used to normalize the antioxidant enzyme analyses (Bradford, 1976) [45].

Superoxide dismutase (SOD) activity was determined using the method based on pyrogallol auto-oxidation, as described by Dieterich et al. [46]. Catalase (CAT) activity was measured based on the rate of hydrogen peroxide decomposition, following the protocol proposed by Aebi [47]. Glutathione S-transferase (GST) activity was assessed by the rate of NADPH oxidation, according to the methodology of Habig, Pabst and Jakoby [48]. Nitric oxide (NO) production was estimated indirectly by quantifying nitrite and nitrate levels described by Tsikas [49].

Lipid peroxidation was measured based on the concentration of malondialdehyde (MDA) through the reaction with thiobarbituric acid [50]. Protein oxidation was assessed by determining the levels of carbonyl groups present in the pellets as described by Levine et al. [51]. The ferric reducing antioxidant power (FRAP) assay was performed using the methodology described by Benzie and Strain [52]. All analyses were performed in triplicate.

### 2.13. Mineral Microanalysis

The analysis of mineral content in cardiac tissue was performed as described by Ladeira et al. [53], using energy-dispersive X-ray spectroscopy (EDS) coupled to a scanning electron microscope (SEM; JEOL, JSM-6010LA, Akishima, Tokyo, Japan) equipped with a Silicon Drift Detector X-ray detector.

Small fragments of the heart (n = 5 per group—number adjusted to make the evaluation among the experimental groups comparable, considering the total number of groups included in the study) were previously dehydrated in an oven and coated with a thin layer of evaporated carbon (Quorum Q150 T, East Grinstead, West Sussex, UK). EDS microanalysis was performed at 350× magnification, with an acceleration voltage of 20 kV and a working distance of 10 mm.

The proportions of the elements calcium (Ca), sodium (Na), potassium (K), magnesium (Mg), iron (Fe), copper (Cu), zinc (Zn), and manganese (Mn) were quantified.

### 2.14. Determination of Ca^*2+*^-ATPase, Na^+^/K^+^-ATPase, and Mg^*2+*^-ATPase Activities

Fragments of the left ventricle (50 mg) were homogenized in Tris-HCl buffer (0.1 M, pH 7.4) and centrifuged at 1500× *g* for 10 min at 5 °C. The resulting supernatant was used for quantification of Ca^2+^, Na^+^/K^+^, and Mg^2+^ATPase activities, according to previously described methodologies [54,55,56]. The enzymatic reaction was initiated by adding ATP (0.01 M) as substrate, allowing the release of inorganic phosphate. After the incubation period, the reaction was stopped with 220 μL of a chilled 10% trichloroacetic acid (TCA) solution, followed by centrifugation under the same conditions. The resulting supernatant was used to determine phosphorus content, employing a commercial biochemical kit (Bioclin, Belo Horizonte, MG, Brazil), according to the manufacturer’s instructions. The remaining pellet was reserved for total protein quantification using the Bradford method [45]. The enzymatic activity of ATPases was expressed in micrograms of phosphorus released per hour and normalized by the total protein content determined by the Bradford method, in order to compensate for variations in protein concentration among samples.

### 2.15. Histological Analyses

Fragments of the left ventricle (n = 6 per group) were fixed in 10% formalin solution for 24 h. Subsequently, they were dehydrated in increasing concentrations of ethanol (70%, 80%, 90%, and 100%) and embedded in 2-hydroxyethyl methacrylate (Historesin^®^, Leica Microsystems, Nussloch, Germany). Histological sections with a thickness of 3 μm were obtained, selecting one section every ten, and stained with toluidine blue. The slides were mounted with Entellan^®^ (Merck, Darmstadt, Germany) [57].

Histological analysis was performed using a photomicroscope (Olympus BX53, Tokyo, Japan) with a 20× objective lens. For each slide, ten images were captured, and the areas and diameters of cardiac fibers were quantified using Image Pro-Plus^®^ software, version 4.5 (Media Cybernetics, Rockville, MD, USA). In addition to the morphometric assessment, a qualitative analysis of the cardiac microstructure was conducted.

### 2.16. Statistical Analyses

Data were expressed as mean ± standard deviation (SD). Normality of distributions was assessed using the Shapiro–Wilk test. Comparisons among groups within the same experimental condition (intragroup) were performed using analysis of variance (ANOVA), followed by Newman Keuls multiple comparison test. For comparisons between conditions with and without RE within each group, Student’s *t*-test for independent samples was applied. All analyses considered a significance level of 5% (*p* < 0.05). The partial eta squared (η^2^) was used to measure the effect size. Statistical analysis and graph preparation were performed using GraphPad Prism software, version 10.1.2 (GraphPad Prism Inc., La Jolla, CA, USA).

## 3. Results

### 3.1. Proximate Composition of Camu-Camu

The powdered camu-camu used in this study showed a high carbohydrate content, corresponding to approximately 95% of the total composition (Table 2).

### 3.2. Concentration of Total Phenolic Compounds, Vitamin C Content and Antioxidant Activity by DPPH, ABSTS, and FRAP in Powdered Camu-Camu Pulp

Analysis of the bioactive composition of camu-camu showed 46.26 ± 1.49 mg gallic acid equivalents (GAE) per gram of sample of total phenolic compounds. Regarding the ascorbic acid content, camu-camu showed approximately 6.47 ± 0.12 mg per gram of sample (Table 3). Antioxidant capacity was evaluated by three different methods. The DPPH free radical assay showed an average value of 419.64 ± 6.41μM Trolox equivalents per gram of sample, while the antioxidant capacity evaluated by the ABTS method was 335.48 ± 2.76 μmol Trolox equivalents per gram. The reducing activity, determined by the FRAP assay, was 155.42 ± 2.49 μmol Trolox equivalents per gram of sample (Table 3).

### 3.3. Mineral Content in Powdered Camu-Camu

Powdered camu-camu pulp showed higher concentration for sodium (34.0 mg/100 g), followed by potassium (24.0 mg/100 g), calcium (9.0 mg/100 g), phosphorus (8.0 mg/100 g), and magnesium (4.0 mg/100 g) (Table 4).

### 3.4. Murinometric and Food Intake Measurements

No differences were observed among the experimental groups in terms of murinometric measurements and food intake (*p* > 0.05) (Table 5).

### 3.5. Antioxidant Enzymes and Oxidative Markers

**Figure 2:**
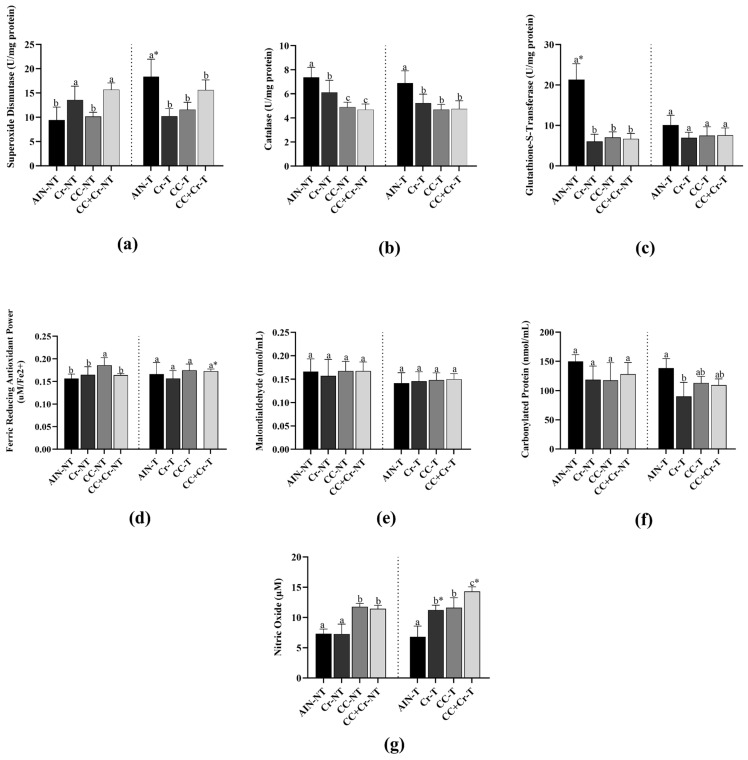
Activities of antioxidant enzymes and oxidative stress markers in the left ventricle. AIN-NT: untrained standard diet group; AIN-T: trained standard diet group; Cr-NT: untrained creatine-supplemented group; Cr-T: trained creatine-supplemented group; CC-NT: untrained camu-camu-supplemented group; CC-T: trained camu-camu-supplemented group; CC + Cr-NT: untrained creatine and camu-camu supplemented group; CC + Cr-T: trained creatine and camu-camu supplemented group. (**a**) Superoxide Dismutase; (**b**) Catalase; (**c**) Glutathione-S-Transferase; (**d**) Ferric Reducing Antioxidant Power; (**e**) Malondialdehyde; (**f**) Carbonylated Protein and (**g**) Nitric Oxide. Different letters indicate significant differences according to ANOVA, followed by the Newman-Keuls test, and Student’s *t*-test for independent groups (*).

SOD activity was higher in AIN-T compared to AIN-NT (*p* < 0.01; η^2^ = 0.71), which indicated that RE, in the absence of supplementation, was able to induce an enzymatic antioxidant response in the left ventricle. Among the untrained groups, Cr-NT and CC + Cr-NT showed the highest SOD activities, with values higher than those observed in AIN-NT and CC-NT (*p* < 0.01; η^2^ = 0.70). On the other hand, among the trained groups, only AIN-T showed increased SOD activity (*p* < 0.01; η^2^ = 0.70), whereas Cr-T, CC-T, and CC + Cr-T presented similar activities among themselves and lower than AIN-NT (*p* > 0.05) (Figure 2a).

CAT activity showed a pattern similar to that of SOD. In the untrained groups, AIN-NT exhibited the highest CAT levels, greater than those of Cr-NT, CC-NT, and CC + Cr-NT (*p* < 0.001; η^2^ = 0.94). Although CC-NT and CC + Cr-NT did not differ from each other (*p* > 0.05), Cr-NT showed higher enzymatic activity than them (*p* < 0.01; η^2^ = 0.94). Among the trained groups, AIN-T presented elevated CAT activity compared to the others (*p* < 0.001; η^2^ = 0.61). However, the supplemented groups (Cr-T, CC-T, and CC + Cr-T) did not differ from each other (*p* > 0.05) (Figure 2b).

Regarding GST activity, a distinct pattern was observed. AIN-NT showed higher GST activity compared to AIN-T (*p* < 0.05; η^2^ = 0.82). Among the untrained groups, AIN-NT maintained the highest values (*p* < 0.001; η^2^ = 0.90), while the supplemented groups (Cr-NT, CC-NT, and CC + Cr-NT) exhibited similar activity levels among themselves, all lower than the control (*p* > 0.05). Among the trained groups, no differences in GST activity were observed (*p* > 0.05) (Figure 2c).

Among the untrained animals, CC-NT presented the highest FRAP values (*p* < 0.05; η^2^ = 0.50). However, among the trained groups, no differences in total antioxidant capacity were observed (*p* > 0.05). The comparison between CC + Cr-T and CC + Cr-NT showed an increase only in the trained group (*p* < 0.05; η^2^ = 0.61) (Figure 2d).

No differences in MDA levels were observed among groups, regardless of supplementation or RE, indicating no changes in lipid oxidative stress (*p* > 0.05) (Figure 2e).

Also, no differences in PC concentration were observed among the untrained groups (*p* > 0.05). However, among the trained groups, Cr-T showed a lower concentration compared to AIN-T (*p* < 0.01; η^2^ = 0.60). The other trained groups that received supplements (CC-T and CC + Cr-T) did not differ from each other (*p* > 0.05) (Figure 2f). Finally, AIN-NT and Cr-NT showed the lowest NO concentrations, while CC-NT and CC + Cr-NT exhibited higher concentrations (*p* < 0.001; η^2^ = 0.86). Among the trained animals, CC + Cr-T stood out with the highest NO concentrations, even surpassing its corresponding untrained group (CC + Cr-NT) (*p* < 0.001; η^2^ = 0.85). In contrast, AIN-T showed the lowest NO levels among the trained groups (*p* < 0.001; η^2^ = 0.82) (Figure 2g).

### 3.6. Mineral Microanalysis

**Figure 3:**
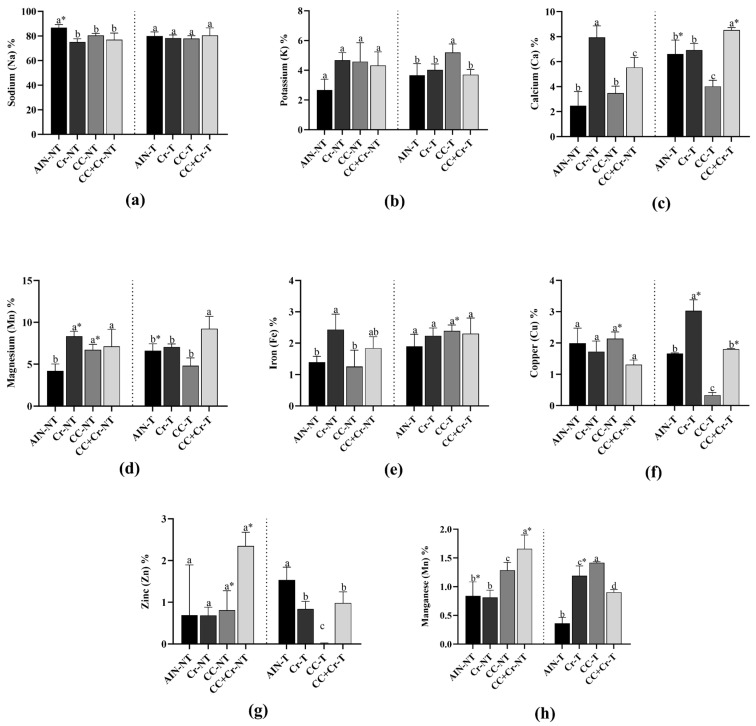
Mineral content in the left ventricle. AIN-NT: untrained standard diet group; AIN-T: trained standard diet group; Cr-NT: untrained creatine-supplemented group; Cr-T: trained creatine-supplemented group; CC-NT: untrained camu-camu-supplemented group; CC-T: trained camu-camu-supplemented group; CC + Cr-NT: untrained creatine and camu-camu supplemented group; CC + Cr-T: trained creatine and camu-camu supplemented group. (**a**) Sodium; (**b**) Potassium; (**c**) Calcium; (**d**) Magnesium; (**e**) Iron; (**f**) Copper (**g**) Zinc and (**h**) Manganese. Different letters indicate significant differences according to ANOVA, followed by the Newman-Keuls test, and Student’s *t*-test for independent groups (*).

The analysis of mineral concentrations in the left ventricle revealed no differences in sodium levels among the supplemented groups (Cr, CC, or CC + Cr), regardless of training (*p* > 0.05). However, the untrained control group (AIN-NT) showed higher sodium concentration compared to the other untrained groups (*p* < 0.01; η^2^ = 0.66), as well as to its trained counterpart (AIN-T) (*p* < 0.01; η^2^ = 0.63) (Figure 3a).

Regarding potassium, no differences were observed among the untrained groups (*p* > 0.05). However, among the trained groups, the camu-camu supplemented group (CC-T) showed higher potassium concentration compared to the others (*p* < 0.01; η^2^ = 0.64) (Figure 3b).

The Cr-NT group showed the highest levels of calcium among the untrained groups, while AIN-NT and CC-NT exhibited the lowest contents (*p* < 0.001; η^2^ = 0.89). Among the trained groups, the highest concentration was observed in CC + Cr-T, whereas CC-T showed the lowest values (*p* < 0.001; η^2^ = 0.90). Comparisons between trained and untrained groups revealed increased calcium content in AIN-T and CC + Cr-T compared to their respective untrained groups (*p* < 0.01, η^2^ = 0.84; *p* < 0.001, η^2^ = 0.91, respectively) (Figure 3c).

Magnesium levels were lowest in AIN-NT compared to the other untrained groups (*p* < 0.01; η^2^ = 0.73). Among the trained groups, the CC + Cr-T group had the highest levels compared to the other groups (*p* < 0.01; η^2^ = 0.79). Comparative analysis between trained and untrained groups showed an increase in magnesium in AIN-T compared to their respective untrained groups (*p* < 0.02; η^2^ = 0.76). However, Cr-NT and CC-NT groups were higher among untrained animals (*p* < 0.01, η^2^ = 0.64; *p* < 0.05, η^2^ = 0.69, respectively) (Figure 3d).

Iron concentration varied among the untrained groups, with Cr-NT standing out by showing the highest levels compared to AIN-NT and CC-NT (*p* = 0.01; η^2^ = 0.64). Among the trained groups, no differences were observed (*p* > 0.05), although the iron concentration in CC-T was increased compared to its untrained counterpart (CC-NT) (*p* = 0.01; η^2^ = 0.71) (Figure 3e).

Copper concentration did not differ among the untrained groups (*p* > 0.05). However, among the trained groups, Cr-T showed the highest concentration, while CC-T had the lowest one (*p* < 0.001; η^2^ = 0.98). Additionally, increased copper levels were observed in Cr-T and CC + Cr-T (*p* < 0.01, η^2^ = 0.84; *p* < 0.01, η^2^ = 0.89, respectively), and decreased content in CC-T compared to their respective untrained groups (*p* < 0.001; η^2^ = 0.98) (Figure 3f).

For zinc, no differences were found among the untrained groups, although CC + Cr-NT showed numerically higher values (*p* > 0.05). Among the trained groups, the control group (AIN-T) exhibited the highest concentration, while CC-T showed the lowest (*p* < 0.001; η^2^ = 0.90). In the comparison between trained and untrained groups, CC-NT and CC + Cr-NT showed higher zinc concentrations compared to their respective trained counterparts (*p* < 0.05, η^2^ = 0.78; *p* < 0.01, η^2^ = 0.89, respectively) (Figure 3g).

Regarding manganese, CC + Cr-NT showed the highest levels, followed by CC-NT (*p* < 0.01; η^2^ = 0.83). Among the trained groups, CC-T and Cr-T presented significantly higher concentrations compared to the others (*p* < 0.001; η^2^ = 0.96). Additionally, increased manganese content was observed in Cr-T (*p* < 0.05; η^2^ = 0.70), and decreased content in AIN-T and CC + Cr-T compared to their respective untrained groups (*p* < 0.05, η^2^ = 0.72; *p* < 0.01, η^2^ = 0.88, respectively) (Figure 3h).

### 3.7. Content of Ca^2+^-ATPase, Na^+^/K^+^-ATPase, and Mg^2+^-ATPase Activities

**Figure 4:**
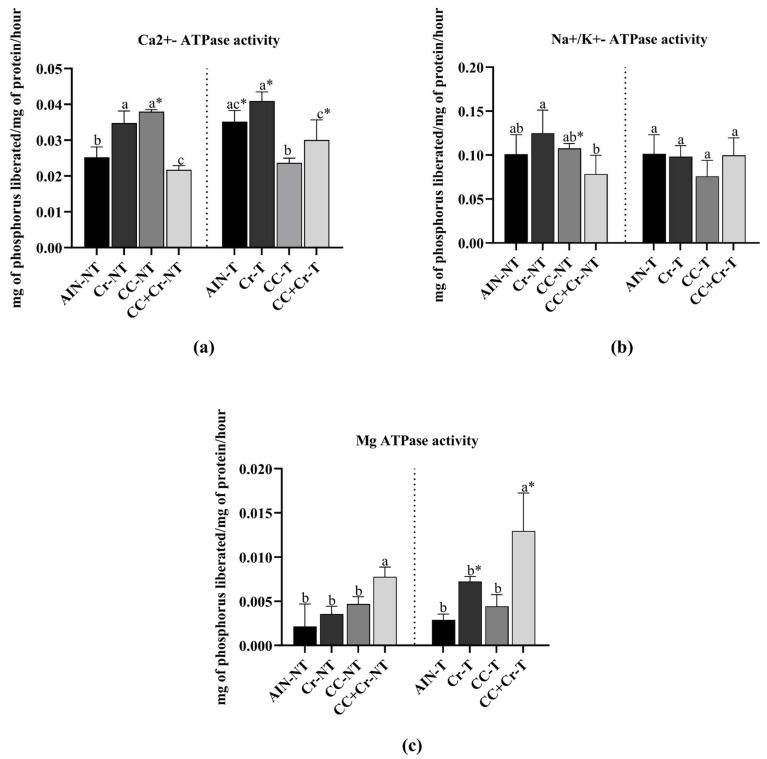
Activities of Ca^2+^-ATPase, Na^+^/K^+^-ATPase, and Mg^2+^-ATPase. AIN-NT: untrained standard diet group; AIN-T: trained standard diet group; Cr-NT: untrained creatine-supplemented group; Cr-T: trained creatine-supplemented group; CC-NT: untrained camu-camu-supplemented group; CC-T: trained camu-camu-supplemented group; CC + Cr-NT: untrained creatine and camu-camu supplemented group; CC + Cr-T: trained creatine and camu-camu supplemented group. (**a**) Ca^2+^-ATPase; (**b**) Na^+^/K^+^-ATPase and (**c**) Mg^2+^-ATPase. Different letters indicate significant differences according to ANOVA, followed by the Newman-Keuls test, and Student’s *t*-test for independent groups (*).

Cr-NT and CC-NT showed higher Ca^2+^-ATPase activity compared to AIN-NT, while CC + Cr-NT demonstrated a reduction (*p* < 0.001; η^2^ = 0.94). Among the trained animals, Cr-T and CC + Cr-T exhibited higher Ca^2+^-ATPase activity compared to CC-T (*p* < 0.01; η^2^ = 0.76), although they did not differ from AIN-T (*p* > 0.05). When comparing the same groups with and without training, increased enzymatic activity was observed in AIN-T, Cr-T, and CC + Cr-T compared to their respective untrained groups (AIN-NT, Cr-NT, and CC + Cr-NT) (*p* < 0.01, η^2^ = 0.79; *p* < 0.05, η^2^ = 0.60; *p* < 0.05, η^2^ = 0.54, respectively). However, in the groups supplemented only with camu-camu (CC), activity was higher in the untrained group (*p* < 0.001; η^2^ = 0.98) (Figure 4a).

Na^+^/K^+^-ATPase activity showed a reduction in CC + Cr-NT compared to Cr-NT (*p* < 0.05; η^2^ = 0.50), with no differences observed in relation to AIN-NT and CC-NT (*p* > 0.05). No differences were identified among the trained groups (*p* > 0.05), a similar result was observed in sodium content. However, in the comparison between the same groups with and without training, decreased enzyme activity was observed in CC-T compared to CC-NT (*p* < 0.01; η^2^ = 0.67) (Figure 4b).

Regarding Mg^2+^-ATPase, CC + Cr-NT exhibited higher activity compared to the other untrained groups (*p* < 0.01; η^2^ = 0.77). Among the trained groups, the activity of this enzyme was higher in CC + Cr-T compared to the others (*p* < 0.01; η^2^ = 0.79), similar to the magnesium content. Additionally, both Cr-T and CC + Cr-T showed increases compared to their respective untrained counterparts (*p* < 0.01, η^2^ = 0.90; *p* < 0.01, η^2^ = 0.51, respectively) (Figure 4c).

### 3.8. Histological Analysis

**Figure 5:**
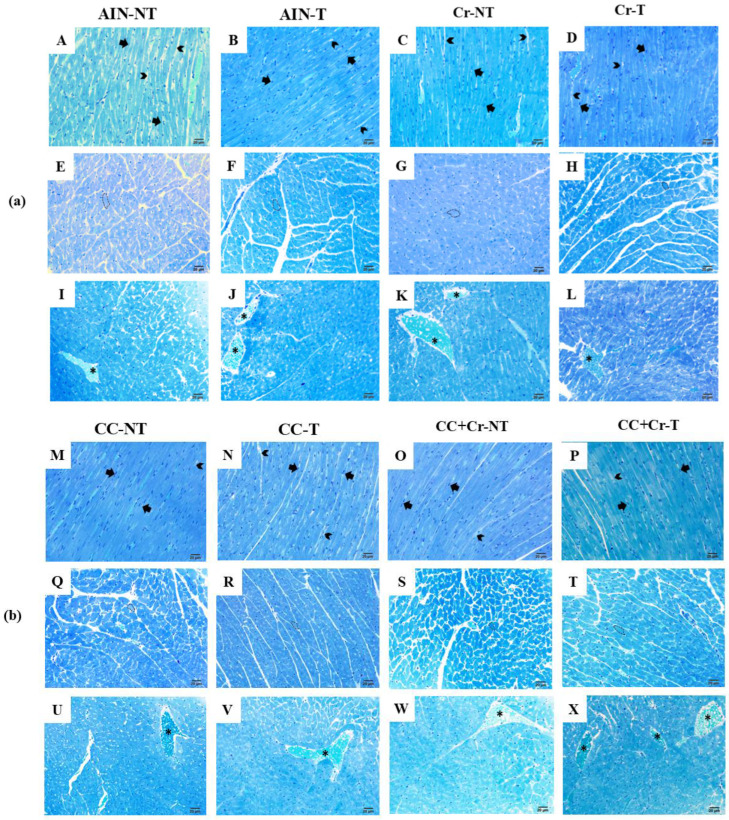
(**a**) (A–L) Representative photomicrographs of the left ventricle stained with toluidine blue (scale bar: 20 µm). Black arrows: cardiomyocytes; arrowheads: extracellular matrix; dotted regions: cardiac muscle fiber area in the cross-section; asterisks: hyperemia. AIN-NT: untrained standard diet group; AIN-T: trained standard diet group; Cr-NT: untrained creatine-supplemented group; Cr-T: trained creatine-supplemented group. (**b**). (M–X) Representative photomicrographs of the left ventricle stained with toluidine blue (scale bar: 20 µm). Black arrows: cardiomyocytes; arrowheads: extracellular matrix; dotted regions: cardiac muscle fiber area in the cross-section; asterisks: hyperemia. CC-NT: untrained camu-camu-supplemented group; CC-T: trained camu-camu-supplemented group; CC + Cr-NT: untrained creatine and camu-camu supplemented group; CC + Cr-T: trained creatine and camu-camu supplemented group.

Qualitative analysis of the left ventricle demonstrated that all experimental groups preserved myocardial architecture, characterized by aligned cardiomyocytes with well-defined central nuclei. No signs of necrosis, hemorrhage, inflammatory infiltrate, or abnormal accumulation of extracellular matrix were observed. Mild hyperemia was also noted in the tissues, possibly related to increased local blood flow, with no evidence of tissue damage. These findings indicate the maintenance of morphostructural integrity of the myocardium in all evaluated groups (Figure 5a,b).

**Figure 6:**
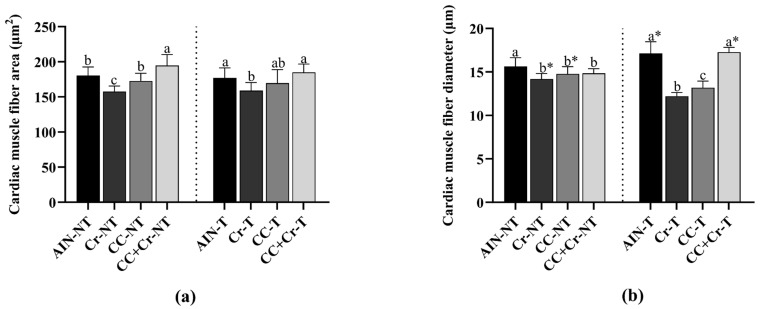
Histomorphometric analysis of cardiac muscle fiber. N = 6. Different letters indicate significant differences according to ANOVA, followed by the Newman-Keuls test, and Student’s *t*-test for independent groups (*). The graphs show (**a**) cardiac muscle fiber area and (**b**) cardiac muscle fiber diameter. AIN-NT: untrained standard diet group; AIN-T: trained standard diet group; Cr-NT: untrained creatine-supplemented group; Cr-T: trained creatine-supplemented group; CC-NT: untrained camu-camu-supplemented group; CC-T: trained camu-camu-supplemented group; CC + Cr-NT: untrained creatine and camu-camu supplemented group; CC + Cr-T: trained creatine and camu-camu supplemented group.

In the histomorphometric analysis of the left ventricle, among the untrained animals, the CC + Cr-NT group had a larger cardiac muscle fiber area, while the Cr-NT group had a smaller area (*p* < 0.001; η^2^ = 0.58) (Figure 6a). In the trained animals, a similar pattern was observed, with the CC + Cr-T group having a larger area and the Cr-T group having a smaller area compared with the AIN-NT and CC + Cr-T groups (*p* < 0.01; η^2^ = 0.66) (Figure 6a). Regarding the diameter of cardiac muscle fibers, in untrained animals, the AIN-NT group had a higher value compared to the Cr-NT group (*p* < 0.01; η^2^ = 0.50) (Figure 6b). Among the trained animals, the CC + Cr-T and AIN-T groups had the largest diameters, while the Cr-T and CC-T groups had the smallest values (*p* < 0.001; η^2^ = 0.88) (Figure 6b). When comparing the same interventions between trained and untrained groups, the diameters of Cr-NT and CC-NT were larger compared to their respective trained groups (*p* < 0.001, η^2^ = 0.79; *p* < 0.001, η^2^ = 0.53, respectively), and the AIN-T and CC + Cr-T groups were larger compared to the untrained groups (*p* < 0.01, η^2^ = 0.50; *p* < 0.001, η^2^ = 0.84, respectively) (Figure 6b).

## 4. Discussion

Physical exercise combined with a balanced diet rich in phenolic compounds (such as those found in camu-camu) is essential for promoting cardiac health and function [58,59]. However, there are no studies in the literature evaluating the association of camu-camu with a supplement, such as creatine, and its effects on the heart. In the present study, we evaluated the effects of supplementation with camu-camu, creatine, and their combination, combined with resistance training, on oxidative balance, ATPase enzyme content, mineral content, and histological parameters in the hearts of Wistar rats.

Our results showed that camu-camu powder had a high content of phenolic compounds and strong antioxidant capacity, as demonstrated by values in the DPPH, ABTS, and FRAP assays. Furthermore, RE promoted increased enzyme activity of the primary antioxidant system in the left ventricle. However, supplementation with creatine, camu-camu, or a combination of these modulated these responses differently, suggesting possible attenuating effects on SOD and CAT enzymes. Furthermore, the combination of exercise and creatine and camu-camu supplementation increased nitric oxide levels and total antioxidant capacity, as well as Mg^2+^-ATPase activity and magnesium content. Histological evaluation revealed preserved cardiac microstructure in all groups, indicating the absence of tissue injury.

Regarding the proximate composition of the camu-camu pulp powder, we verified a high carbohydrate content (94.90%) and low contents of lipids (1.24%), proteins (0.42%), fiber (0.80%), and moisture (3.29%). The high carbohydrate value can be attributed to the addition of cassava starch as an excipient, a carbohydrate frequently used in the spray drying technique, which possibly influenced this result [60,61]. The low moisture content is expected, since this process involves water removal under high temperatures in a short period of time [62].

From a functional perspective, camu-camu exhibited high antioxidant activity, as demonstrated by the elevated levels of total phenolic compounds (46.26 ± 1.49 mg GAE/g) and the results obtained in the DPPH (419.64 ± 6.41 μM Trolox/g), ABTS (335.48 ± 2.76 μmol TE/g), and FRAP (155.42 ± 2.49 μmol TE/g) assays, in addition to the significant presence ascorbic acid (6.47 + 0.12 mg/g), reinforcing its antioxidant potential.

As for mineral content, higher concentrations of sodium, potassium, and calcium were observed, with 34 mg/100 g, 24 mg/100 g, and 9.0 mg/100 g, respectively. Although in small amounts, they still contribute to the nutritional profile of camu-camu.

In this study, the parameters evaluated in the left ventricle indicate that RE was able to induce an adaptive antioxidant response, demonstrated by an increased SOD and CAT enzyme activity in the AIN-T group compared to its untrained control (AIN-NT). These findings are consistent with the literature, as regular physical exercise generates adaptive responses to oxidative stress, enhancing endogenous antioxidant defense [63]. In this context, the controlled production of ROS during training acts as a stimulus for the increased activity of mitochondrial antioxidant enzymes [64]. Among these enzymes, SOD stands out as one of the main components of the primary antioxidant defense system due to its ability to catalyze the dismutation of superoxide radicals into hydrogen peroxide and molecular oxygen [65]. Studies report that physical exercise may induce cardioprotection precisely through the allosteric activation of SOD, contributing to the maintenance of oxidative homeostasis and protecting cardiac tissue against oxidative stress induced damage [66,67].

However, the groups supplemented with creatine, camu-camu, or the combination of both did not exhibit the same pattern. A possible explanation, suggested by previous studies, is that exogenous antioxidant compounds can influence the regulation of endogenous antioxidant enzymes [68,69]. In this context, camu-camu stands out due to its high content of vitamin C and phenolic compounds, such as cyanidin-3-glucoside, which are well-known for their antioxidant properties [12,70]. The intake of these compounds may modulate the expression or activity of antioxidant enzymes [68]. Similarly, studies have shown that supplementation with vitamins C and E reduced the mRNA expression of antioxidant enzymes, including SOD1, GPx, and CAT, in individuals subjected to physical exercise [71,72].

In addition to its ergogenic role, creatine also exhibits antioxidant properties, contributing to the neutralization of reactive oxygen species [73]. Moreover, creatine can increase the expression of myogenic regulatory factors (MRFs) and insulin-like growth factor-1 (IGF-1) mRNAs [23,74,75], as well as elevate phosphocreatine (CrP) stores [23,76]. These effects support cellular energy metabolism and may be associated with its ability to protect mitochondrial integrity, including mitochondrial DNA (mtDNA), thereby preventing oxidative damage [23]. Thus, both isolated supplementation and the combination of creatine and camu-camu may have reduced the need for activation of the primary antioxidant response in the trained groups.

Although our data do not directly assess molecular mechanisms, it is possible that supplementation provided sufficient antioxidant support to modulate the activity of endogenous enzymes, alternatively, the influence of dosage cannot be ruled out.

Regarding the GST enzyme, a distinct pattern was observed: the AIN-NT group showed higher activity compared to AIN-T, while the supplemented groups, both trained and untrained, exhibited lower values. Thus, the higher GST activity in the AIN-NT group may be related to the periodic performance of maximal load tests (as described in Section 2.9.), which, despite the absence of a regular training protocol, may have induced a compensatory activation of enzymes such as GST. In untrained rats, these stimuli generated by maximal load testing may have led to increased GST activity as a protective response mechanism.

The analysis of total antioxidant capacity, evaluated by the FRAP method, revealed that the CC-NT group showed the highest values among the untrained groups. This result may be associated with the high concentration of vitamin C and phenolic compounds present in camu-camu, as observed in this study (155.42 ± 2.49 µmol TE/g of sample). These compounds act as electron donors, neutralizing ROS [10,77]. Among the trained groups, although no differences were observed, the increase in FRAP values in the CC + Cr-T group compared to its corresponding untrained group (CC + Cr-NT) suggests a possible additive effect between physical exercise and combined supplementation with camu-camu and creatine. This finding can be explained by the antioxidant properties of creatine, previously discussed, including its ability to preserve mitochondrial integrity and reduce oxidative damage [78].

The absence of differences in MDA levels among the groups indicates that the interventions did not directly impact lipid peroxidation. However, carbonylated protein levels increased in the AIN-T group compared to the Cr-T group, suggesting that creatine may play a protective role against protein oxidation, possibly due to its ability to preserve mitochondrial integrity and neutralize reactive oxygen species [23,78].

Oxidative balance is known to modulate processes such as cell signaling, mitochondrial function, and excitation-contraction coupling, which are fundamental steps for neuromuscular efficiency [79,80]. Interventions with antioxidants, such as curcumin, have been associated with improvements in markers of muscle performance, endurance, strength, and reaction time [81,82,83,84], suggesting that modulation of the oxidative state may favor adaptations that enhance motor and functional performance. These effects may be reflected in neuromotor performance, as evidenced by Mancini et al. [85], who demonstrated that faster reaction times are correlated with better performance in agility tests in young athletes. This perspective broadens the physiological relevance of our results, indicating that improving oxidative homeostasis may favor not only cardiac health but also overall functional performance.

We observed that supplementation with camu-camu, either isolated or in combination with creatine, promoted increased nitric oxide (NO) concentrations, especially in the CC + Cr-T group. This finding can be partially explained by the effects of physical exercise, which, when performed regularly, stimulates NO production through increased blood flow and activation of the nitric oxide synthase (NOS) enzyme [86]. Endogenous NO production occurs through the conversion of L-arginine into NO and L-citrulline, a reaction catalyzed by NOS. Thus, the increased NO may suggest a possible beneficial effect on endothelial function; however, this cannot be confirmed, as no complementary measures of vascular function were evaluated [86,87,88].

Furthermore, the compounds present in camu-camu, such as vitamin C and polyphenols, may contribute to an increased NO bioavailability by reducing oxidative stress, thereby protecting NO from degradation by ROS [89]. Additionally, these compounds—especially anthocyanins and procyanidins—stimulate the PI3K/Akt pathway, enhancing the expression and synthesis of endothelial nitric oxide synthase (eNOS), which promotes greater NO production [90]. Creatine, in turn, may contribute to this process by sparing arginine, the amino acid precursor of NO, as well as through its mitochondrial antioxidant action, which can support the stability and functionality of the NO pathway [23]. Thus, the observed results suggest a possible effect between physical exercise and the combined supplementation of camu-camu and creatine on NO production.

Regarding the mineral microanalysis of the left ventricle, changes related to RE and different supplementations were observed. Sodium, potassium, and calcium are important minerals for cardiac excitability and contraction [19,21]. Sodium content did not show differences among the trained groups. During physical exercise, the increased influx of sodium into cardiomyocytes through sodium channels under physiological conditions is compensated by the action of the Na^+^/K^+^-ATPase [91,92]. Therefore, the absence of variation among the trained groups suggests that the supplementation used did not directly interfere with the regulation of this electrolyte in response to exercise, corroborating the findings of Na^+^/K^+^ATPase activity, which also showed no changes in the trained groups.

A higher potassium content was observed only in the CC-T group. This mineral is important for the repolarization of cardiac cells and for maintaining the electrical excitability of the myocardium [93]. However, there is currently no direct association between camu-camu supplementation and increased potassium content in cardiac tissue.

Calcium concentrations were higher in the CC + Cr-T group. This result is interesting, as physical exercise leads to increased expression of calcium-regulating proteins involved in the release and uptake of this mineral, thereby enhancing cardiac contraction and excitability [8,94]. Thus, supplementation with antioxidant-rich camu-camu and creatine may have acted synergistically, reducing oxidative stress, preserving cellular function, and indirectly facilitating the regulation of intracellular ions [23,95]. Additionally, creatine can improve calcium uptake by the sarcoplasmic reticulum, increasing the availability of ATP for the Ca^2+^ATPase pump. This mechanism occurs due to the creatine-phosphocreatine system, which supports local ATP regeneration, which could explain both the increase in calcium in the supplemented groups and the increase in Ca^2+^ATPase activity observed in the Cr-T group [96]. Furthermore the MDA results reinforce this interpretation, since the lack of significant differences indicates lower lipid peroxidation, a condition that may compromise ATPase pump activity [97,98].

An increased concentration of magnesium, a mineral important for the regulation of cardiac contractility, ATP production, and ATPase enzyme activity, was observed in the CC + Cr-T group [99,100]. During physical exercise, there is a higher energy demand, which intensifies ATP utilization [101]. Since magnesium is essential for the formation of the functional Mg^2+^ATP complex (a cofactor for various enzymes in glycolysis and the citric acid cycle), this increased metabolic activity may justify the greater recruitment and accumulation of this mineral [102,103]. In this context, combined supplementation with creatine and camu-camu may have potentiated this effect. Creatine acts in ATP resynthesis, while camu-camu, rich in bioactive compounds with antioxidant properties, may have favored the preservation of mitochondrial integrity and, consequently, the regulation of magnesium in the myocardium. These findings corroborate greater Mg^2+^ATPase activity in the CC + Cr-T group, suggesting that increased Mg availability contributed to enzyme efficiency [23,104,105,106].

The microminerals iron, copper, zinc, and manganese, cofactors of antioxidant enzymes such as SOD and iron for CAT, showed heterogeneous results and did not follow the pattern observed for the enzymes in this study [107,108]. Copper concentration, a cofactor of SOD, was higher in the CR-T and CC + Cr-T groups, while the CC-T group exhibited the lowest levels. However, this variation in copper content was not proportionally reflected in SOD activity, which was increased only in the AIN-T group. This difference suggests that although copper is essential for SOD function, its isolated presence does not guarantee higher enzymatic activity, reinforcing the role of physical exercise [63,109].

The reduction in zinc levels observed in the supplemented and trained groups may be related to lower SOD levels, considering that this mineral acts as a cofactor of Cu/Zn-SOD [110,111]. Manganese, another cofactor of SOD, showed higher levels in the CC-T and CR-T groups [112]. Despite this, SOD activity was not higher in these groups, suggesting that the presence of this mineral alone does not determine enzyme activity. These findings indicate that variations in tissue levels of these minerals may not directly translate into functional changes, since enzymatic activity also depends on other regulatory factors. In this case, for example, the presence of exogenous antioxidants (such as camu-camu and creatine) may have attenuated this demand, contributing to the lower SOD activity observed [23,70].

Qualitative histological analysis demonstrated preservation of morphology in all evaluated groups. These findings indicate that, regardless of the intervention (RE, supplementation with camu-camu, creatine, or their combination), no tissue damage occurred, reinforcing the safety of the strategies used in the experimental model. The identified hyperemia may be associated with increased blood flow induced by RE. During muscular activity, there is an elevation of blood supply to active tissues to meet the higher local metabolic demand [113]. Thus, even in animals not submitted to regular training sessions but exposed to periodic maximal load tests, as in the present experimental protocol, hyperemia may occur, which explains the histological observation without representing tissue injury.

Regarding the histomorphometric analysis, an increased diameter of cardiac muscle fibers was observed in the CC + Cr-T group, suggesting physiological hypertrophy. However, we acknowledge that this interpretation is based on morphological parameters, and further analyses are required to assess markers of hypertrophic signaling.

In this sense, regular RE can promote a specific type of physiological concentric cardiac hypertrophy, characterized by the thickening of the left ventricular wall and an increase in cardiomyocyte width [8]. Unlike pathological concentric cardiac hypertrophy, the physiological form is not associated with the presence of apoptosis or fibrosis in the heart; on the contrary, it may promote potentially beneficial adaptations [7].

Additionally, creatine combined with camu-camu potentiated the effects of RE in the present study. Although the mechanisms are unclear, creatine is involved in ATP resynthesis and has been associated with cardiac contractile function [25,114,115]. Camu-camu, through its bioactive compounds, possesses antioxidant and anti-inflammatory properties that, together, may have contributed to this outcome [70,104].

In this context, it is worth emphasizing that all the changes observed in this study are beneficial and reflect physiological adaptations, thereby reinforcing the safety and effectiveness of the interventions, given that the animals were healthy and not subjected to pathological induction.

This study has some limitations that should be acknowledged. The use of an experimental animal model, although suitable for controlled physiological analysis, limits the direct extrapolation of the results to human populations. The relatively small sample size may limit statistical power, and the exclusive inclusion of male Wistar rats restricts the extrapolation of results to possible sex differences in response to RE and supplementation. The use of only males aimed to minimize variability resulting from hormonal fluctuations during the estrous cycle, which can affect oxidative stress, the inflammatory response, and cardiovascular adaptation. Furthermore, this choice sought to ensure comparability with previous investigations that adopted similar experimental designs. Furthermore, the study period was relatively short, which may not have captured long-term adaptations in cardiac function and redox balance. Future studies should consider increasing the sample size, explore different doses and durations of camu-camu and creatine supplementation, investigate potential molecular signaling pathways involved in cardiac adaptations, and assess functional outcomes such as cardiac output, performance and cardiac parameters assessed by echocardiography. Translational research in humans, particularly in individuals engaged in resistance training, is warranted to confirm whether the combined use of camu-camu and creatine modulates cardiac parameters and exerts similar antioxidant effects.

## 5. Conclusions

Powdered camu-camu showed a promising nutritional composition, which contributed to its high concentration of total phenolic compounds and strong antioxidant activity.

RE altered the activity of antioxidant enzymes, with increased levels of SOD and CAT. In contrast, creatine and camu-camu supplementation in trained animals attenuated the activity of these enzymes but was able to increase antioxidant capacity, Mg^2+^ATPase activity, tissue magnesium content, and nitric oxide levels, suggesting potentially beneficial adaptations. In addition, the combination of supplementation with RE promoted a greater cardiac muscle fiber diameter, without pathological changes (in all experimental groups). In this sense, the data suggest that RE combined with camu-camu and creatine supplementation may be an effective strategy, contributing to cardiovascular health.

Future studies should investigate the underlying molecular mechanisms involved in the modulation of cardiac parameters, evaluate different doses and durations of supplementation, and determine whether similar effects occur in humans through translational or clinical studies.

## Figures and Tables

**Table 1:** Composition of experimental diets.

Compounds	AIN-93M (g/kg of Diet)
Corn starch	449.2
Albumin ^1^	156.5
Maltodextrin	155.0
Sucrose	100.0
Soybean oil	40.0
Cellulose	50.0
Mineral mixture	35.0
Vitamin mix	10.0
L-cystine	1.8
Choline bitartrate	2.5
Creatine *	-
Camu-camu *	-

AIN-93M: standard maintenance diet for rodents; ^1^ albumin based on 76.68% protein content. * creatine and camu-camu were supplemented.

**Table 2:** Proximate composition of camu-camu powder pulp and total fiber content.

Proximate Composition	%
Moisture	3.29
Lipids	1.24
Total ash	0.15
Proteins	0.42
Total carbohydrates	94.90
Fiber	0.80
TEV kcal	398.74
ED	3.98

Values expressed on a dry basis. TEV: total energy value; ED: energy density.

**Table 3:** Concentration of total phenolic compounds, ascorbic acid content and antioxidant capacity of camu-camu.

Parameter	Value/g Sample
Total phenolic (mg GAE)	46.26 ± 1.49
Ascorbic acid (mg)	6.47 ± 0.12
DPPH (µM Trolox)	419.64 ± 6.41
ABTS (µmol TE)	335.48 ± 2.76
FRAP (µmol TE)	155.42 ± 2.49

Data expressed as mean ± standard deviation (SD). DPPH: 2,2-diphenyl-1-picrylhydrazyl. ABTS: 2,2′-azino-bis (3-ethylbenzothiazoline-6-sulfonic acid); FRAP: Ferric Reducing Antioxidant Power.

**Table 4:** Mineral content in camu-camu powder pulp.

Minerals	Content (mg/100 g)
Phosphorus (P)	8.0 ± 0.00
Potassium (K)	24.0 ± 0.57
Calcium (Ca)	9.0 ± 1.00
Magnesium (Mg)	4.0 ± 0.00
Copper (Cu)	0.0177 ± 0.00
Iron (Fe)	0.2836 ± 0.11
Zinc (Zn)	0.1196 ± 0.04
Manganese (Mn)	0.0951 ± 0.00
Sodium (Na)	34.0 ± 1.00

Data expressed as mean ± standard deviation (SD).

**Table 5:** Murinometric measurements and food intake of trained and untrained rats.

Parameters	AIN-NT	AIN-T	Cr-NT	Cr-T	CC-NT	CC-T	CC + Cr-NT	CC + Cr-T
Weight gain (grams)	79.42 ± 8.09	78.08 ± 7.81	81.66 ± 13.53	80.36 ± 7.42	79.10 ± 11.22	83.14 ± 7.52	85.23 ± 15.19	80.85 ± 12.37
WC gain (centimeters)	1.05 ± 0.12	1.25 ± 0.21	1.50 ± 0.41	1.35 ± 0.88	1.03 ± 0.21	1.61 ± 0.67	1.28 ± 0.32	0.96 ± 0.47
BMI (g/cm^2^)	0.62 ± 0.04	0.63 ± 0.03	0.63 ± 0.05	0.65 ± 0.07	0.68 ± 0.01	0.69 ± 0.02	0.66 ± 0.05	0.69 ± 0.04
TFI (grams)	801.40 ± 33.93	841.30 ± 95.97	718.60 ± 49.30	812.90 ± 101.30	762.20 ± 62.96	811.80 ± 60.82	757.30 ± 106.00	820.30 ± 59.15
FEC	9.98 ± 0.51	9.32 ± 1.84	11.24 ± 2.13	9.60 ± 2.09	10.52 ± 2.14	10.58 ± 1.89	11.16 ± 0.94	9.93 ± 0.98

Data are expressed as mean ± SD. N = 6. Statistical analysis was performed by ANOVA separately for untrained and trained groups, followed by the Newman-Keuls post hoc test. Statistical differences among groups within the same training condition were indicated by different letters in the same row. When no significant difference was observed, values were presented without letters. Comparisons between the same treatment in trained and untrained groups were conducted using Student’s *t*-test, with significant differences indicated by an asterisk (*). AIN-NT: untrained standard diet group; AIN-T: trained standard diet group; Cr-NT: untrained creatine-supplemented group; Cr-T: trained creatine-supplemented group; CC-NT: untrained camu-camu-supplemented group; CC-T: trained camu-camu-supplemented group; CC + Cr-NT: untrained creatine and camu-camu supplemented group; CC + Cr-T: trained creatine and camu-camu supplemented group; WC: waist circumference; BMI: body mass index; TFI: total food intake; FEC: feed efficiency coefficient.

## Data Availability

The data will be shared upon reasonable request to the corresponding author due to ethical restrictions related to the use of animals in research.
